# Time-of-Flight PET/CT Imaging of Ga-68-Dotatate: Normal Pattern, SUV Quantification, and Differences from Non-Time-of-Flight Imaging

**DOI:** 10.1055/s-0044-1786529

**Published:** 2024-05-07

**Authors:** Matthew Clifton Miller, Avani T. Bansal, Daniel Wingard, Maria Liza Lindenberg, Derek J. Stocker, Stephen Adler, Kalpna Prasad

**Affiliations:** 1 School of Medicine, Uniformed Services University of the Health Sciences, Bethesda, Maryland, United States; 2 The Potomac School, McLean, Virginia, United States; 3 USA Radiology, WRNMMC, Bethesda, Maryland, United States; 4 Molecular Imaging Program, NCI, NIH, Washington, DC, United States; 5 Nuclear Medicine, WRNMMC, Bethesda, Maryland, United States; 6 SAIC PET Physicist, Chevy Chase, Maryland, United States

**Keywords:** NET, dotatate, TOF, PET/CT

## Abstract

**Purpose** The biodistribution of gallium-68-dotatate (Ga-68-dotatate) and standardized uptake values (SUVs) using non-time-of-flight (TOF) positron emission tomography/computed tomography (PET/CT) cameras is well established. However, with the eventual retirement of older PET cameras and their replacement with newer, highly sensitive TOF PET/CT cameras, where SUV _max_ measurements are reportedly higher, updated knowledge of normal SUV _max_ range is needed and, to our knowledge, not previously reported. Our objectives are as follows:

To establish normal Ga-68-dotatate TOF SUV _max_ database for common structures and to aid the visual detection of abnormalities objectively.

To compare SUV _max_ values using the TOF and non-TOF algorithms.

**Methods** Fifty consecutive patients referred routinely to our nuclear medicine service (20 men, 30 women; median age 55 years) with presumed neuroendocrine tumors underwent Ga-68-dotatate scans on a PET-CT camera having capability of reconstructing both TOF/non-TOF images. Region of interests (ROIs) were drawn around 24 normal structures as well as the primary lesion with abnormal radiotracer uptake and SUV _max_ was measured. The same ROI was analyzed using both algorithms simultaneously and both TOF and non-TOF SUV _max_ values were compared.

**Results** Twelve hundred ROIs were evaluated. Non-TOF Ga-68-dotatate uptake in normal structures was in alignment with previously published studies. As compared to non-TOF, TOF images had better target to background ratios visually. TOF SUV _max_ was higher for all structures except for lung and brain. TOF SUV _max_ was more than double in adrenals/uncinate process of the pancreas; approximately 1.8 times in abnormal lesions, lymph nodes, pineal gland; and greater than 1.5 times in thyroid, breast, and pancreatic head.

**Conclusion** Normal database of Ga-68-dotatate TOF SUV _max_ is provided for common structures to aid visual detection of abnormalities objectively. Overall, TOF SUV _max_ measures higher in identical ROIs, with abnormal lesions measuring approximately 1.8 times higher versus non-TOF technology. These findings need to be taken in consideration when comparing patient scans imaged on different PET/CT technologies.

## Introduction

Surveillance, epidemiology, and end results data from 1973 to 2012 shows that even though neuroendocrine tumors (NET) are rare tumors, their incidence is gradually rising over the years. 1 Accordingly, there has been an increased need for improved methods of imaging these tumors. Somatostatin receptors (SSTR) are high-affinity G protein membrane receptors that are preferentially expressed in neuroendocrine cells. These SSTRs have five subtypes with subtype 2/SSTR2 representing higher density in most NETs as well as lung, breast, and prostate cancer. 2 3 4 These SSTR2 receptors proved to be a viable target for SST analogues. In the early 1990s, diethylene triamine penta-acetic acid-octreotide (pentetreotide or DTPA-octreotide) was developed and radiolabeled with Indium-111 (In-111). Its binding to SSTR was imaged via gamma camera and had a proven sensitivity of greater than 75%. 5 In-111 octreotide's main detractors were its extended time from injection to imaging (up to 48 hours), high radiation dose, and lower image resolution. In 2016, gallium-68 dotatate (Ga-68-dotatate) was approved by the Food and Drug Administration for use with positron emission tomography (PET)/computerized tomography (CT) cameras. Ga-68-dotatate was proven to be highly sensitive, specific, and accurate (94%), and versus In-111 octreotide, Ga-68-dotatate PET/CT had 100 times the affinity for SST2 receptors, a lower overall radiation dose (0.08 vs. 0.02 millisieverts/megabecquerel) (mSv/MBq) and required less than two hours to complete the entire study. 6 7 Ga-68-dotatate uptake is determined by visual analysis and assisted by semiquantitative measures such as maximum standardized uptake value (SUV _max_ ). SUV is a surrogate for concentration of radiotracer activity in a lesion and is calculated using voxel values of activity concentration per unit volume (kilobecqueral [kBq]/milliliters [mL]) normalized to the available concentration of radiotracer injected in the patient's body (injected radiotracer activity in Mbq divided by patient weight in kilograms [kg]). It is calculated using the following formula.



SUV _max_ represents the maximum voxel value within a region of interest (ROI) and is most commonly used clinically as it is not prone to observer variation. It helps in objectively differentiating abnormal activity from physiological distribution, and comparing it to reference organs such as liver and spleen for interpretation. 8 9 10 Due to these advancements, Ga-68-dotatate PET/CT has rapidly become the preferred imaging modality for evaluating NETs. 6 11 12 Just as SST analogues and new radiopharmaceuticals continue to emerge, new imaging technology and more sensitive and specific cameras continue to unfold such as the newer time-of-flight (TOF) cameras.

### Conventional Non-TOF PET/CT Cameras versus TOF PET/CT Cameras

In conventional PET imaging, a positron released from the radiotracer annihilates with an electron and releases two photons in opposite directions along a 180-degree line of response (LOR). During the PET scan, the released photons from the annihilation striking the opposing detectors at nearly the same time are included for image construction, and the slight time difference of their detection allows computers to determine their approximate location along the LOR. Multiple intersecting LORs are then used to determine a more precise location of the annihilation event, thereby the site of radiotracer accumulation. Ultimately, using CT attenuation correction and the anatomical images obtained from the CT, the exact location in the body where the annihilation event occurred is determined. 13 Conventional PET/CT cameras (a.k.a. the non-TOF cameras) detect the slight time difference of annihilation photons striking the detectors within nanoseconds. They utilize all the coincident photons striking their detectors within a coincidence time window of about 10 nanoseconds for scintillation detection and image generation.

In 2006, a more advanced technology that could detect even shorter time differences of photon detection was introduced, which has progressively improved over the years. These advancements in technology have introduced newer scanners, such as those employing lutetium-based crystal scintillators or digital systems utilizing silicon photomultipliers. These innovations allow for a more precise recording of the difference in coincident photon arrival times, decreasing the coincidence time window to approximately 0.6 nanoseconds. Consequently, this enhancement facilitates superior localization of the annihilation event, resulting in improved signal-to-noise ratio and temporal resolution, and more sensitive imaging. This technique is used by various manufacturers who give it different names and is referred to as TOF imaging by GE (Boston, Massachusetts, United States) PET/CT camera system. This TOF PET/CT technology allowing for more precise measurement of the time difference between photon detection down to the pico-second is becoming increasingly sought after. 13 This additional ultra-short time difference data shortened the spread of activity allowing the detection algorithms to decrease the spread of activity along the LOR, provide even more precise. This additional ultra short time difference data allows the detection algorithms to shorten the spread of activity along the LOR, providing even more precise localization, better target to background ratio, improved image quality, and more accurate detection of abnormal lesions. This decrease in spread of activity is one of the reasons for higher SUV _max_ noted with TOF imaging, especially for smaller lesions. With the eventual retirement of older non-TOF PET cameras and their replacement with newer, highly sensitive TOF PET/CT cameras, where SUV _max_ measurements are reportedly higher, updated knowledge and database of normal ranges of SUV _max_ are needed.

To our knowledge, TOF SUV _max_ in normal structures on Ga-68-dotatate scans has not been previously reported. Additionally, awareness of how much the TOF SUV _max_ differs from the non-TOF value is required for adequate scan comparisons, while the different technologies coexist, and for follow-up examinations over a period when camera technology may change between scans. Our study objectives were as follows:

1. To establish normal average TOF SUV _max_ values for common structures on Ga-68-dotatate PET/CT
2. To compare SUV _max_ values obtained using the TOF and non-TOF algorithms.

## Materials and Methods

Retrospective analysis of 77 consecutive clinically referred Ga-68-Dotatate scans was performed. Only the scans performed on the new PET-CT camera that had the capability of creating images using both TOF and non-TOF image reconstruction capability were included in the study. Only 50 patients underwent imaging on the camera with both reconstruction capabilities and were included in the analysis. The remaining 27 patients did not undergo scanning using both reconstruction capability due to camera availability and thus did not have comparison TOF versus non-TOF imaging and were excluded. Since there were no specific characteristics of the excluded 27 patients, the generalizability of the results is not affected.

Upon submission of this study's objectives and design, the parent institution's Department of Research Programs determined that this project did not require Institutional Review Board approval as per 32 Code of Federal Regulation 219.102 and Department of Defense Instruction 3216.02 and approved the conduction of the study as a quality improvement project.

### Study Population

Inclusion criteria for the retrospective analysis were as follows: (1) Any patient with known/presumed NET clinically imaged on the PET-CT camera having both the TOF and non-TOF reconstruction capability, (2) age older than 18 years, (3) not pregnant. Retrospective analysis of 77 consecutive Ga-68-dotatate patient scans from 2016 to 2019 was performed out of which 50 patients (20 men and 30 women; median age 55 years; range 18–88; median weight 88.5 kg, see Table 1 ) were selected. Twenty patients were excluded as they underwent imaging with only TOF reconstruction without having comparison non-TOF analysis. Their exclusion should thus not affect the generalizability of this study as the lack of dual analysis was the only known variable. All patients were imaged using identical PET imaging protocol.

**Table 1:** Patient characteristics

Study characteristics *n* = 50	Inclusion criteria: 1) Known/presumed NET using standard imaging 2) Age older than 18 years 3) Not pregnant	
	Mean	Median
Age (years)	53.4	55
Male:female	20:30	–
Weight (kg)	85.9	88.5
Dose (mCi)	4.6	4.9
Injection to scan time (minutes)	61.0	60

Abbreviation: NET, neuroendocrine tumor.

### Radiopharmaceutical

Unit doses of Ga-68-dotatate for each clinical patient were manufactured as per standard manufacturing protocol in commercial pharmacies following strict nuclear pharmacy regulations. 14 These clinical doses were received by a nuclear pharmacist and inspected for colorless and particulate free appearance. The doses were injected, and patients were imaged using identical PET imaging protocols.

### Ga-68-Dotatate PET/CT Imaging Protocol

Patients were asked to fast 2 hours prior to injection. A minimum of 24 hours after short-acting SST analogues or just prior to the next dose of long-acting SST analogues was required. Patients were then injected with a prescribed dose of 185 MBq / 5 millicurie (mCi) of Ga-68-dotatate. Average dose was 4.6 mCi, and median was 4.9 mCi. The average interval between injection to scan time was 61 minutes. Whole body imaging was performed from vertex to mid-thigh without contrast. Imaging was performed on a Siemens Biograph mCT (Munich, Germany) (see Table 2 ).

**Table 2:** PET-CT camera characteristics for dotatate imaging

Camera	Siemens Biograph mCT
	64 detector, 128-slice (max) CT and time-of-flight capable PET-CT camera
**CT scout image**	120 kVp 35mA Over 10.4 seconds
**Whole-body CT images**	120 kVp Weight-based mA Pitch 1.0 3 mm slice CT Matrix= 512 × 512 (default
**PET emission scan was performed in 3 dimensions and slice overlap between consecutive bed acquisitions.** **PET images were reconstructed using an ordered subsets expectation maximization algorithm and CT-based attenuation correction**	4 minutes/bed Axial coverage = 22 cm 2 iterations and 21 subsets 3D IR TOF and NTOF Transaxial PET slice-thickness 3 mm (PET slice thickness matches with CT)
**NO oral or IV contrast given**	

Abbreviations: IR, infrared; IV, intravenous; PET/CT, positron emission tomography/computed tomography; TOF, time of flight.

A CT scout image was acquired with 120kVp, 35mA, over 10.5 seconds. Whole-body CT images were acquired at 120kVp, weight-based mA, 1.0 pitch, and 3 mm slices. Maintaining patient position, a PET emission scan was performed in three-dimensional mode and a 5-slice overlap between consecutive bed acquisitions at 4 minutes/bed. PET images were reconstructed using an ordered subsets expectation maximization algorithm using 2 iterations and 21 subsets per manufacturer recommendations and CT-based attenuation correction. 15 Transaxial PET slice thickness was 3mm on the Siemens mCT (Munich, Germany). PET slice thickness was matched to CT slice thickness.

### Image Analysis

Ga-68-dotatate uptake was measured via SUVs calculated using the following equation as described previously:



where *r* is the radioactivity activity concentration [kBq/ml] measured by the PET scanner within a ROI, *a* ′ is the decay-corrected amount of injected radiolabeled FDG [kBq], and *w* is the weight of the patient, which is used a surrogate for a distribution volume of tracer. 16 Maximum SUV (SUV _max_ ) within a ROI is the most commonly utilized clinically. It represents the maximum voxel value within that ROI and remains unaffected by observer variation.

**Fig. 1:**
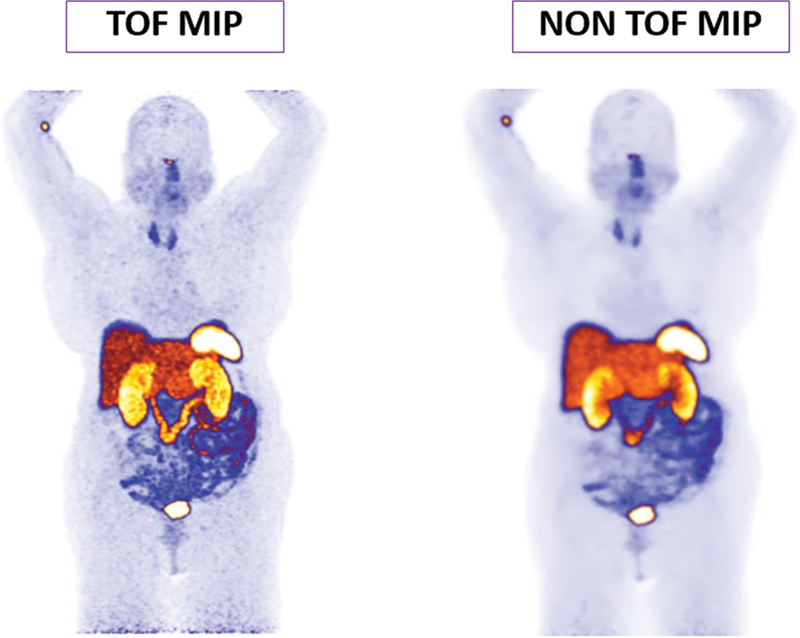
Maximum intensity projection (MIP) image of patient with time of flight (TOF) and non-TOF.

**Fig. 2:**
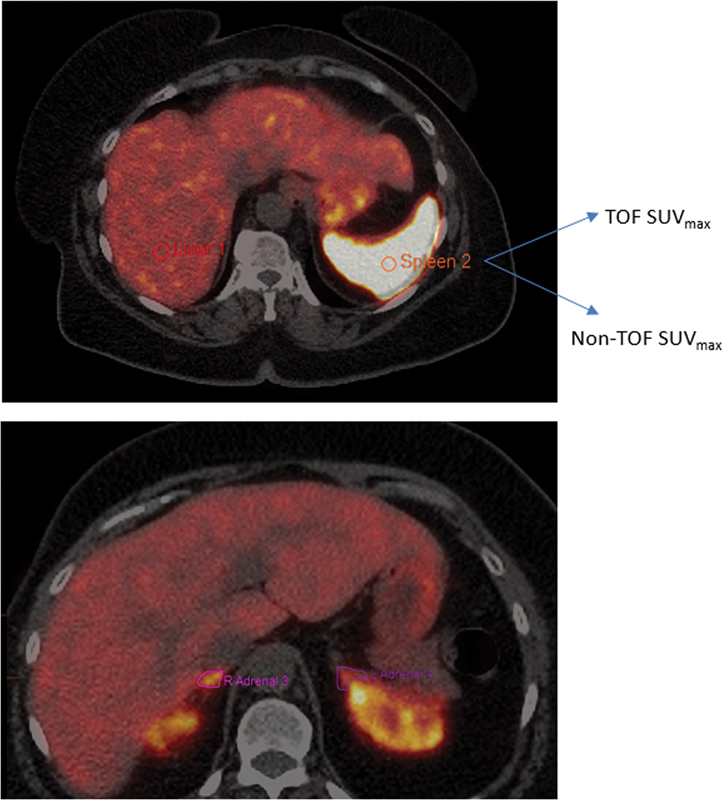
A one centimeter square (cm2) region of interest (ROI) was drawn on an anatomically normal appearing area of 24 preferentially chosen structures to measure maximum standardized uptake value (SUV _max_ ) SUV _max_ . Same ROI was analyzed using both algorithms and the SUV _max_ were compared. TOF, time of flight.

Images were reviewed by a board-certified nuclear medicine physician and 5th year radiology resident using a Hermes workstation (Hermes Medical Solutions, Stockholm). SUV _max_ was measured on axial images. Each image was processed using TOF and non-TOF reconstruction algorithms ( Fig. 1 ). A semiautomated 1-centimeter square (cm ^2^ ) ROI was drawn on 24 anatomically normal appearing structures ( Table 3 ) to measure SUV _max_ while avoiding any confounding adjacent activity or areas concerning for any possible disease or abnormality on CT and when unavoidable, a manually contoured region was drawn ( Fig. 2 ). The 1 cm ^2^ ROI was placed on the area with the highest appearing uptake visually while avoiding adjacent organs. The same ROI was analyzed using both algorithms and the SUV _max_ was compared. Bone marrow uptake was measured at the 5th lumbar vertebral body, blood uptake was measured at the left atrium, and lung uptake was measured at the right upper lobe. Manual ROIs were also drawn for the suspected tumor lesion.

**Table 3:** ROI list

24 Analyzed structures
Adrenal (left)
Adrenal (right)
Blood pool
Bone marrow
Brain
Breast
Fat
Lesion (abnormal uptake)
Liver
Lung
Lymph node
Muscle
Myocardium
Pancreas body
Pancreas head
Pancreas tail
Pancreas uncinate
Pineal
Pituitary
Renal cortex
Salivary gland
Spleen
Thyroid
Urine

Abbreviation: ROI, region of interest.

### Statistical Analysis

Average values were calculated for all data such as age, radiopharmaceutical dose, and imaging time. Data was shown as an average of SUV _max_ with a 95% confidence interval (CI) for both TOF and non-TOF. A paired t-test was performed with a threshold of *p* -value less than 0.05 to determine significance.

## Results

### Physiologic Biodistribution of Ga-68-Dotatate in Normal Structures with TOF/Non-TOF Algorithm

**Fig. 3:**
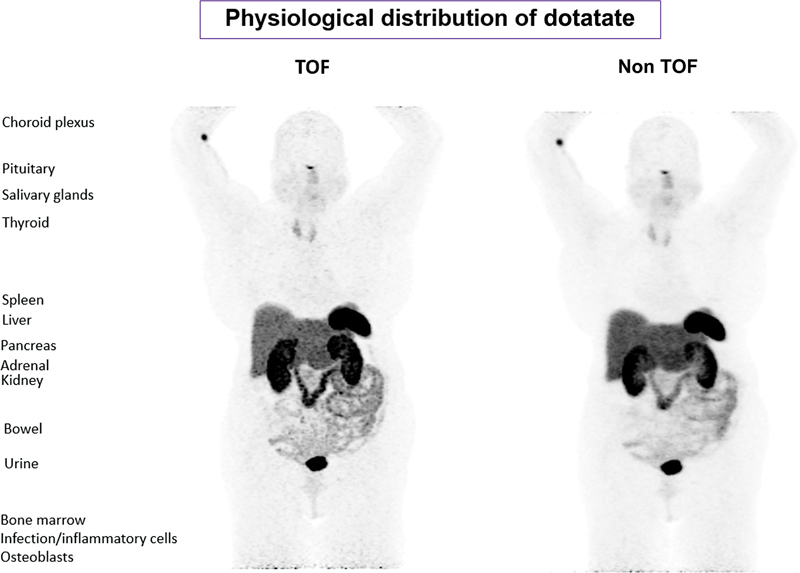
Physiologic distribution of dotatate in normal structures. TOF, time of flight.

**Fig. 4:**
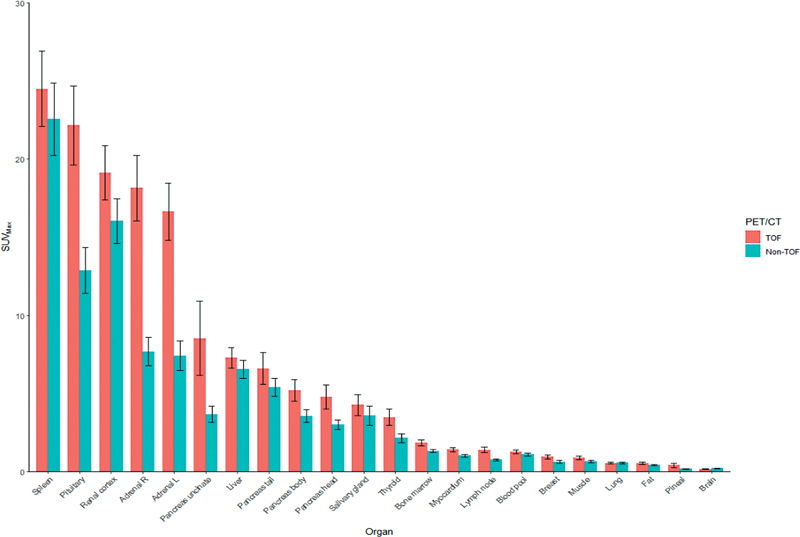
Maximum standard uptake value (SUV _max_ ) for different organs. Each bar depicts the average SUV _max_ , across scans, split by organ and positron emission tomography/computed tomography (PET/CT) scan type. Error bars are 95% confidence intervals within each subset. TOF, time of flight.

**Fig. 5:**
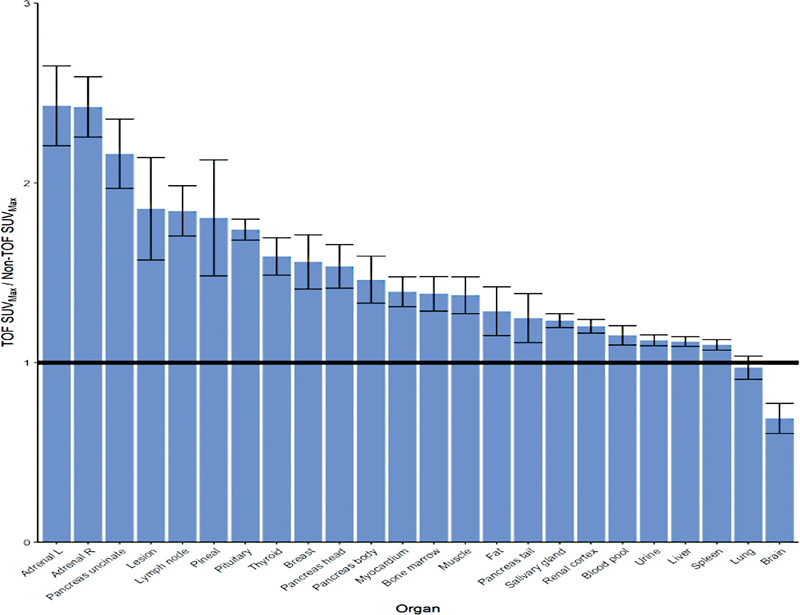
Average ratio of the time-of-flight (TOF) and non-TOF maximum standard uptake value (SUV _max_ ) for each organ. Error bars are the 95% confidence intervals for each set of ratios. The solid horizontal line indicates a ratio of one (i.e., equivalent SUV _max_ ).

A total of 1200 ROIs were evaluated. Fig. 3 visually depicts the distribution of Ga-68-dotatate in a typical study patient with both TOF and non-TOF cameras. Fig. 4 depicts the mean and 95% CI for the 24 selected ROIs for both TOF and non-TOF. Table 4 provides the numerical data for Fig. 4 . Fig. 5 details the average ratio of the TOF and non-TOF SUVmax for each organ.

**Table 4:** Average TOF SUV _max_ and non-TOF in identical ROI in normal structures and sample lesion

Organ ( *n* = 50)	TOF SUV max (average)	TOF SUVmax 95% confidence interval	Non-TOF SUVmax (average)	Percentage higher in TOF	*p* -Value
Adrenal (left)	16.63	14.82–18.45	7.40	2.428	< 0.0001
Adrenal (right)	18.14	16.04–20.23	7.67	2.422	< 0.0001
Blood pool	1.27	1.14–1.39	1.10	1.151	< 0.0001
Bone marrow	1.85	1.65–2.05	1.32	1.381	< 0.0001
Brain	0.16	0.13–0.19	0.22	0.688	< 0.0001
Breast	0.94	0.80–1.08	0.62	1.559	< 0.0001
Fat	0.54	0.46–0.63	0.42	1.285	< 0.0001
Lesion (abnormal uptake)	49.92	34.01–65.83	34.70	1.855	< 0.0001
Liver	7.28	6.63–7.94	6.54	1.116	< 0.0001
Lung	0.55	0.49–0.61	0.56	0.971	< 0.5
Lymph node	1.39	1.22–1.56	0.75	1.843	< 0.0001
Muscle	0.88	0.78–0.99	0.64	1.374	< 0.0001
Myocardium	1.40	1.27–1.54	1.03	1.393	< 0.0001
Pancreas body	5.19	4.49–5.88	3.54	1.460	< 0.0001
Pancreas head	4.78	4.01–5.55	3.00	1.534	< 0.0001
Pancreas tail	6.60	5.57–7.62	5.39	1.247	< 0.0014
Pancreas uncinate	8.53	6.15–10.91	3.66	2.161	< 0.0001
Pineal	0.40	0.26–0.54	0.17	1.804	< 0.0001
Pituitary	22.15	19.63–24.67	12.87	1.738	< 0.0001
Renal cortex	19.12	17.40–20.85	16.02	1.201	< 0.0001
Salivary gland	4.26	3.59–4.93	3.57	1.232	< 0.0001
Spleen	24.49	22.08–26.90	22.54	1.097	< 0.0001
Thyroid	3.48	2.95–4.01	2.14	1.590	< 0.0001
Urine	79.22	62.95–95.50	70.83	1.123	< 0.0001

Abbreviations: ROI, region of interest; SUVmax, maximum standardized uptake value; TOF, time of flight.

The urinary system had the highest observed activity for both TOF/non-TOF algorithms with a TOF SUV _max_ of 79.22 (95% CI ± 15.91, *p* <0.0001). Non-TOF urinary system SUV _max_ measured 70.83 ( *p* < 0.0001). The high SUV _max_ values were due to the excretion of the radiotracer into the renal collection system and bladder. The highest normal physiologic uptake for TOF and non-TOF was the spleen with a TOF SUV _max_ of 24.49 (95% CI ± 2.41, *p* < 0.0001). Non-TOF spleen SUVmax measured 22.54 ( *p* <0.0001).

Ga-68-dotatate uptake in normal structures on non-TOF images was observed in the following decreasing order: spleen, renal cortex, pituitary, adrenals, liver, pancreas tail, uncinate pancreas, salivary gland, pancreas body, pancreas head, thyroid, bone marrow, blood, myocardium, lymph node, muscle, breast, lung, fat, brain, pineal.

Ga-68-dotatate uptake in normal structures on TOF images was observed in the following decreasing order: spleen, pituitary, renal cortex, adrenals, uncinate pancreas, liver, pancreas except uncinate, salivary gland, thyroid, bone marrow, myocardium, lymph node, blood, breast, muscle, lung, fat, pineal gland, and brain ( Fig. 4 )

### Spectrum of Uptake in Lesions TOF versus Non-TOF

All 50 patients included in this study had suspected NET lesions and each ROI was assessed using TOF/non-TOF algorithms. TOF images had better tumor to background ratios visually. Non-TOF SUV _max_ average lesion uptake was 34.7 as compared to TOF SUV _max_ at 49.2 ( *p* <0.0001).

### TOF versus Non-TOF SUV _max_ Comparison and Statistical Significance

As compared to non-TOF SUV _max_ , TOF SUV _max_ was higher for all structures except for lung and brain. All TOF versus non-TOF SUV _max_ values were significantly different ( *p* <0.0001) with the exception of the lung ( *p* < 0.5). The lack of statistical significance in this one organ system is unclear and requires further scientific exploration.

As compared to non-TOF SUV _max_ , TOF SUV _max_ measured more than double in adrenals, and uncinated pancreas; approximately 1.8 times in concerning lesions, lymph nodes, pineal gland; and greater than 1.5 times in thyroid, breast, and pancreatic head. The spleen to liver ratio was approximately 3.36 with TOF versus 3.44 with non-TOF.

## Discussion

Ga-68-dotatate uptake via non-TOF was in relative alignment with previous studies. 17 18 19 We expand upon the previous knowledge of Ga-68-dotatate distribution using a TOF capable camera system.

### Study Limitations

Given the retrospective nature of this study, it is inherently prone to certain limitations. The study population were all military members or their dependents, which may not be representative of the general population. The writers of this study have no known reasons why they would not be generalizable versus a civilian cohort. Our study included the full extent of available Ga-68-dotatate studies from 2016 to 2019 ( *n* = 77) with minimal exclusion criteria (final *n* = 50). Thus, the results of this study should have a high level of generalizability. All imaging protocols are in alignment with community standards and are clearly provided, further increasing generalizability. The study could have been improved, however, through the inclusion of a National Electrical Manufacturers Association (NEMA) phantom as a standardized reference value. NEMA phantoms provide a standardized and reproducible test environment. They have well-defined geometries and material compositions, ensuring consistency across different imaging systems and study settings. This standardization allows for more reliable comparisons between different imaging modalities, protocols, or facilities. This and all referenced previous Ga-68-dotatate studies have not utilized a NEMA phantom to our knowledge. Avenues for future research should include a standardized NEMA phantom as it would add further credibility and validity to their findings.

### Validity of Non-TOF Uptake Data

Overall biodistribution and overlap are good between this study and previous studies. 17 18 19 As compared to Shastry et al's non-TOF study 18 whose patient population included NET patients with negative scans and no evidence of disease in a 2-year follow-up period, we have similar number of patients (50 vs. 42) and median age (55 vs. 50). The male/female ratio was 30:20 (vs. 15:27). Overall biodistribution and overlap are good between our studies. Our study yielded the same top 5 SUV _max_ organs as Shastry et al with the exception of ordering. The pituitary was the third highest uptake (SUV _max_ 12.87) and was fifth in Shastry et al (SUV _max_ 7.6). Our study's top five organ systems were the spleen (22.54), renal cortex (16.02), pituitary (12.87) adrenals (7.6), and liver (6.54). Shastry et al's top five organ systems were the spleen (27.9), renal cortex (13.6), adrenals (12.5), liver (8.2), and then the pituitary (7.6). The potential difference in uptake between the pituitary SUV _max_ in these studies needs further investigation. It could potentially be due to numerous reasons to include scanner differences or ROI size/location.

### Ga-68-Dotatate Distribution in TOF versus Non-TOF

Utilizing newer TOF PET technology yielded mean TOF SUV _max_ values ( Fig. 5 ) that were consistently higher for all structures except for lung and brain. As compared to non-TOF SUV _max_ , TOF SUV _max_ measured more than double in adrenals, and uncinate process of the pancreas; approximately 1.8 times in concerning lesions, lymph nodes, pineal gland; and greater than 1.5 times in thyroid, breast, and pancreatic head ( Fig. 5 ). TOF suspected lesions measured approximately 1.8 times higher versus non-TOF lesions ( *p* <0.0001).

Patients may obtain PET/CT scans using different facilities and cameras that may show different radiotracer uptake values. Awareness of these differences is useful in scan interpretation. Additionally, readers may now have a potential database of SUV _max_ ranges for the TOF cameras, especially for the PET/CT cameras used in this study.

This study also compared TOF/non-TOF values for abnormal lesions in our population of 50 patients. SUV _max_ non-TOF values aligned with previous studies that characterized typical lesional uptake of Ga-68-dotatate via non-TOF systems, most recently being Moradi et al in 2016. The average SUV _max_ abnormal lesion uptake for non-TOF suspected tumors was 34.7 compared to Moradi et al that found mean SUV _max_ values of 29.3 ± 17.6 for 157 hepatic lesions deemed malignant. Moradi et al's data validated the use of SUV analysis in the interpretation of malignant versus benign lesions primarily that SUV _max_ evaluation alone approaches the accuracy of clinical assessment based on multimodal imaging interpretation and follow-up. 17 Furthermore, Moradi et al highlighted that malignant lesions have a statistically significant increase in uptake versus benign lesions in most cases.

The exact cause of these differences is not well documented. It is theorized that for small lesions less than a centimeter in diameter, partial volume effects come into play and TOF provides better partial volume reconstruction thus making the SUV _max_ higher. 8 19 Additionally, if the TOF reconstruction has more iterations than the non-TOF reconstruction, that tends to cause higher SUV _max_ values.

The large increase in SUV _max_ when using TOF technology could lead to clinical misinterpretation of therapy response and subsequent mismanagement. Practitioners should be aware and understand the differences between TOF and non-TOF Ga-68-dotatate SUV _max_ values.

## Conclusion

Overall, TOF SUV _max_ measures approximately 2X higher in identical ROIs versus non-TOF. This is also true of abnormal NET lesions (1.8X). These findings need to be taken in consideration when comparing patient scans imaged on different PET/CT technologies. This is of particular significance in transient populations (such as the US Military, government, and civilian populations) where they are more likely to receive follow-up or surveillance scanning on potentially different technologies. Additionally, as hospital systems continue to maximize their investment in PET scanners, existing scanners that lack TOF-reconstruction will persist for many years into the future. It is of clinical importance that these findings be taken into consideration or there is the potential for misdiagnosis if incorrect assumptions are made regarding the equivalency of SUV _max_ values across technologies.

Our study furthers the clinical knowledge base of Ga-68-dotatate uptake in newer TOF systems by providing a Ga-68-dotatate TOF physiologic distribution pattern, providing a side-by-side comparison to non-TOF data on identical ROIs and lesions. This novel data should improve interpretation accuracy as well as allow relative SUV comparison when a patient's prior scan was from a non-TOF camera, avoiding potential misdiagnoses or medical mismanagement.

These study findings could be further advanced through the development of standardized protocols or software tools that account for differences in reconstruction algorithms and imaging characteristics to ensure reliable comparison of SUV _max_ values across technologies. Furthermore, future research could investigate the impact of differences in SUV _max_ measurements between TOF and non-TOF PET/CT systems on clinical outcomes, such as diagnostic accuracy, treatment response assessment, and patient management decisions. Large-scale prospective studies could be conducted to evaluate whether discrepancies in SUV _max_ values lead to differences in patient outcomes or influence clinical decision-making.
